# Theoretical investigation on a simple turn on fluorescent probe for detection of biothiols based on coumarin unit

**DOI:** 10.3389/fchem.2023.1290745

**Published:** 2023-11-08

**Authors:** Tianhao Ma, He Huang, Yuling Liu, Yongjin Peng

**Affiliations:** ^1^ Affiliated 3rd Hospital, Jinzhou Medical University, Jinzhou, China; ^2^ College of Bio-Informational Engineering, Jinzhou Medical University, Jinzhou, China

**Keywords:** fluorescent probe, biothiols, quantum mechanical, electron transfer, proton transfer

## Abstract

The discovery of a simple and efficient detection method for biothiols would be scientifically significant due to the crucial role of them in various physiological processes. Recently, a simple fluorescent probe, DEMCA-NBSC, based on coumarin fragments, was developed by Ding et al., and provided an efficient way for real-time sensing of biothiols both *in vivo* and vitro. Theoretical insights to the fluorescence sensing mechanism of the probe were provided in this work. Details of the electron transfer process in the probe under optical excitation and the fluorescent character of the probe were analyzed using a quantum mechanical method. All these theoretical results could inspire the development of a highly convenient and efficient fluorescent probe to sense biothiols both *in vivo* and vitro.

## 1 Introduction

Biothiols, including cysteine (Cys), homocysteine (Hcy), and glutathione (GSH), have strong REDOX and nucleophilic properties (Gothland et al., 2023; Liu and Liu, 2023; Pandya et al., 2023; Qu et al., 2023). As the main chemical antioxidants, biothiols protect cells and tissues from endogenous reactive oxygen species (ROS) and free radicals (Mezhnina et al., 2022; Villavicencio-Tejo et al., 2022; Chen et al., 2023). They are involved in information transmission, cell growth and apoptosis, protein formation, immune regulation, and other processes in living systems. Cys can assist in the synthesis of antioxidant GSH and maintain intracellular homeostasis of oxidation and reduction (Takagi et al., 2012; Blasco et al., 2018). It can also take advantage of the reversible oxidation of glutathione disulfide (GSSG) to protect the thiol-containing proteins and enzymes from injury by free radicals, peroxidation, and heavy metals.

Biothiols are involved in many transfer and detoxification processes, including cell growth, REDOX, and so on. The normal level of Cys in the human body (30–200 μmol/L) is essential to maintain the tertiary and quaternary structure of proteins; Cys is also an important source of sulfide in the human metabolism process (Weerapana et al., 2010; Kisty et al., 2023; das Neves et al., 2023; Cao et al., 2023). Excess Cys in the human body could lead to rheumatoid arthritis, Parkinson’s, and Alzheimer’s disease. Cys deficiency, meanwhile, can induce developmental delays in children and cause edema, liver injury, and skin injury (Pang et al., 2020; Wang et al., 2023). The normal serum concentration of Hcy in healthy adults is 9–13 μmol/L. When the concentration of Hcy in serum is higher than 15 μmol/L, hyperhomocysteinemia could be induced (Ganguly and Alam, 2015; Zhong et al., 2023). GSH is the most abundant non-protein mercaptan in cells. The normal concentration *in vivo* is between 1 and 10 mmol/L, and it plays a key role in the control of oxidative stress of the cell apoptosis in a REDOX stable state (Huang et al., 2023; Swiderski et al., 2023). Abnormality in GSH concentration is also observed as a signal for many diseases, such as AIDS, cancer, lung damage, and Parkinson’s disease (Su et al., 2020; Niu et al., 2021).

In view of the importance of biothiols, accurate detection of its concentration and distribution in the organism are important for disease assessment and diagnosis. Fluorescence analysis technology has great potential for concentration monitoring and intracellular imaging of biothiols *in vivo* due to its high sensitivity, simple operation, and low levels of damage to biological samples (Liu et al., 2021; Zhou et al., 2023a; Zhang et al., 2023).

To date, many fluorescent probes have been developed to detect biothiols, although many have a specific response to only one of the biothiols (Long et al., 2022; Kaushik et al., 2023; Si et al., 2023; Tang et al., 2023; Wu et al., 2023). However, there is a close relationship between different biothiols. A change in one biothol in specific cellular metabolic processes may lead to another biothol changing, and many diseases are associated with the combination of two or more biothiols. Therefore, detecting two or more biothiols simultaneously is more valuable for biological research and disease diagnosis (Hao et al., 2022; Hou et al., 2022; Jiang et al., 2022; Li et al., 2022; Ma et al., 2022; Zhou et al., 2023b; Peng et al., 2023; Zhu et al., 2023).

In 2014, Guo et al. reported the first dual-signal fluorescent probe for simultaneous detection of Cys and GSH (Liu et al., 2014). In 2018, Yin’s research group synthesized a functional fluorescent probe through which biothiols such as Cys, Hcy, and GSH could be distinguished by three different emission channels (Yin et al., 2019). In 2019, Song’s research group constructed the first case of a fluorescent probe that can detect Cys, Hcy, GSH, and H_2_S simultaneously within four light-emitting channels (Zhang et al., 2019). Along with the continuous progress of the design concept, the study of fluorescent probes with the ability to detect biothiols simultaneously has become a hot topic in biological and medical field (Raut and Sahoo, 2021; Ding et al., 2023a; Du et al., 2023; Kaushik et al., 2023; Shellaiah and Sun, 2023; Zhang et al., 2023).

Although recently some related work has been done, the application of fluorescent probes for rapid simultaneous detection of biothiols in both vivo and vitro is still a hot topic. Ding et al. successfully designed and developed a simple fluorescent probe, namely, DEMCA-NBSC, based on coumarin fragments, which presented remarkable emission enhancement and exhibited yellow-green fluorescence after the addition of biothiols (Hcy/Cys/GSH), and the intensified emission response was ascribed to biothiols inducing the cleavage of NBSC group to form a fluorescent compound, DEMCA-OH, which exhibited a strong aggregation-induced emission (AIE) property (Ding G. et al., 2023). The rupture of an S-O bond occurred when the probe DEMCA-NBSC contacted with biothiols. DEMCA-OH was then successfully formed and was the sensing mechanism of DEMCA-NBSC for biothiols which has been confirmed and reported according to the literature (Chen et al., 2022; Goswami et al., 2022). The colorimetrical and fluorescent responses to Cys, Hcy, and GSH was confirmed in reference 45.

It is worth mentioning that DEMCA-NBSC exhibits a rapid response (within 3 min), high selectivity, outstanding sensitivity, and lower detection limits (0.236, 0.223, and 0.365 μM for Cys, GSH, and Hcy, respectively) to biothiols. This probe provides an effective tool for the real-time detection of biothiols both *in vivo* and vitro.

Due to the excellent properties, such as simple synthetic procedure, low cytotoxicity, outstanding sensitivity, and high selectivity, the probe DEMCA-NBSC offered an efficient approach for biothiols detection in and out of biological systems, and has a potential application in diagnostics. In this work, the fluorescent mechanism of the probe DEMCA-NBSC and the remaining compound DEMCA-OH were investigated in detail using a quantum mechanical method. The electronic structure analysis on the ground S_0_ and first excited S_1_ states of the probe DEMCA-NBSC revealed the electron transfer process of the probe under optical excitation. By means of electronic structure, intramolecular interaction, IR spectrum, potential energy scan curve, energy surface hopping dynamics, and charge analysis on the remaining compound DEMCA-OH, the typical excited state intramolecular proton transfer (ESIPT) process that caused the fluorescence phenomenon in DEMCA-OH was investigated in detail. The comprehensive analysis on the probe DEMCA-NBSC and remaining compound DEMCA-OH could provide deep insights into the structure-function relationship of the fluorescent probes.

All these theoretical results could provide insights for understanding the fluorescent principle and building highly effective fluorescent probes for detection of biothiols simultaneously in biological samples.

## 2 Conformation search, electronic structure, and fluorescent properties

The processes of the conformation search for DEMCA-OH were as follows.

(1) Using Confab (O'Boyle et al., 2011) to obtain initial conformations of DEMCA-OH;
(2) Conducting batch structural optimization using Crest to invoke the xtb program under the GFN2-xTB method (Bannwarth et al., 2019);
(3) Invoking isostat in the Molclus (Lu, 2021) program to screen out several stable probe conformations with the local lowest free energy;
(4) Using the functional and basis set combination B3LYP, PBE0, M06-2x and CAM-B3LYP/def2-TZVPD in structure optimization and analyzing the corresponding vibrational frequency on the stable probe conformations obtained from step (3) within the ORCA program (Neese, 2018). Zero negative frequency was found within the optimized structure, which confirmed the local minimum energy of the corresponding structure (Ahlrichs and Ahlrichs, 2005; Grimme et al., 2011; Yu et al., 2016; Ghosh et al., 2018). Similar results were obtained in the probe structure optimization using the four functional as B3LYP, PBE0, M06-2x and CAM-B3LYP. For clarity, the results of the CAM-B3LYP/def2-TZVPD combination were used for the following analysis. The wB2GP-PLYP/def2-TZVPD combination was used in single point energy and TDDFT calculation to obtain the free energy with high precision according to the benchmark research (Grimme and Grimme, 2010; Brauer et al., 2014; Grimme and Grimme, 2014; Boese, 2015; Peng et al., 2021; Liu et al., 2022; Lin et al., 2023; Liu et al., 2023). An SMD model was used in the calculation within a solvent environment (Zhou et al., 2015; Martins and Martins, 2019; Zhao et al., 2021; Zhao et al., 2022). Most of the figures in this work were rendered by means of VMD 1.9.3 software (Humphrey et al., 1995) and the analyses were finished by using the Multiwfn 3.8 (dev) code (Chen and Chen, 2012).
(5) The electronic structure and fluorescent properties of probe were obtained based on the DFT and TDDFT results by ORCA program.
(6) The dynamics on the excited state of the probe were conducted using the Newton-X program (Barbatti et al., 2014).

## 3 Results and discussion

**FIGURE 1:**
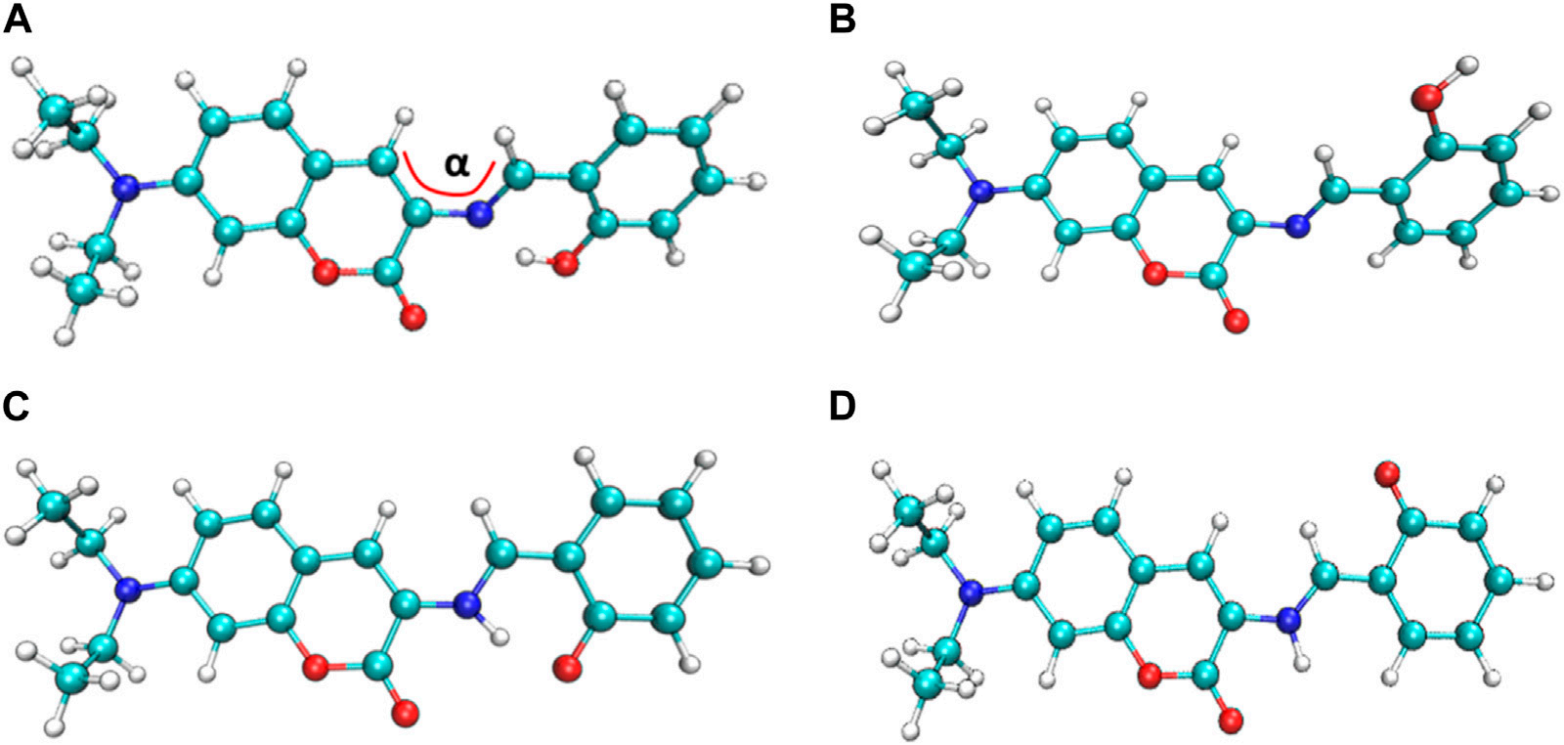
The four most stable probe conformations of probe DEMCA-OH. **(A)** E1 **(B)** E2 **(C)** K1 **(D)** K2.

**FIGURE 2:**
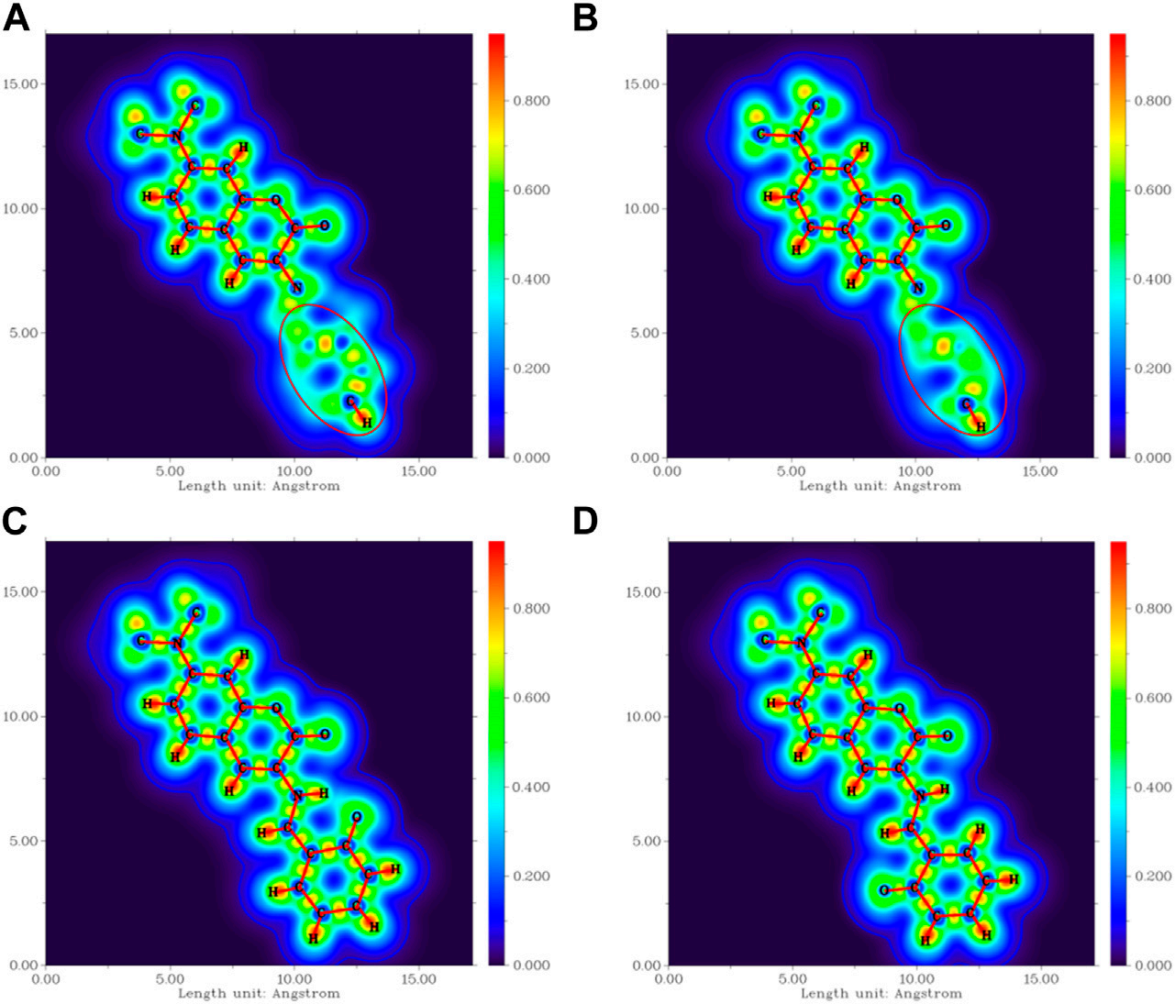
The localized orbital locator projection on the naphthalene ring plane in the four-probe conformation **(A)** E1 **(B)** E2 **(C)** K1 **(D)** K2.

The four most stable conformations of probe DEMCA-OH (named E1, E2, K1, and K2) are shown in Figure 1 and summarized in Table 1. Apart from the two end ethenyl, the whole planar structure is shown in molecule K1 and K2, while there are different dihedral values α between the benzene and naphthalene ring within the molecule E1 and E2. The potential energy scan curve on dihedral angle α is provided in Supplementary Figure S1 for reference. The localized orbital locator projection on the naphthalene ring plane in the four-probe conformation clearly indicated the planar structure in molecule K1 and K2. Meanwhile a larger dihedral value α between the benzene and naphthalene ring within the E2 molecule than E1 molecule is also clearly shown in Figure 2.

**TABLE 1:** Parameters of the four stable probe conformations.

	α	ΔG (kcal/mol) E1 was taken as References
E1	27°	0
E2	42°	4.31
K1	0°	4.60
K2	180°	11.07

**FIGURE 3:**
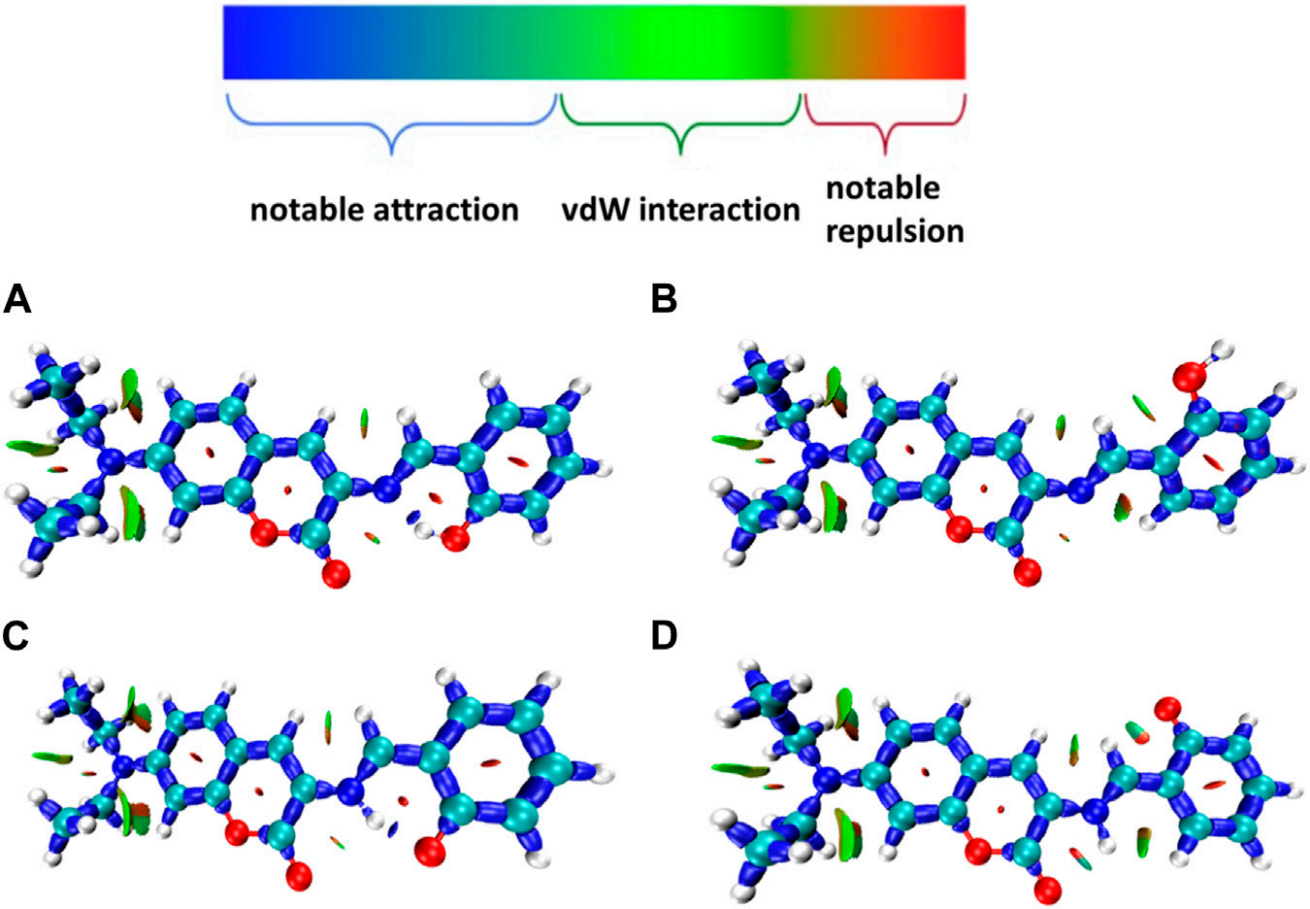
Many interactions between adjacent atoms in four probe molecules **(A)** E1 **(B)** E2 **(C)** K1 **(D)** K2.

**FIGURE 4:**
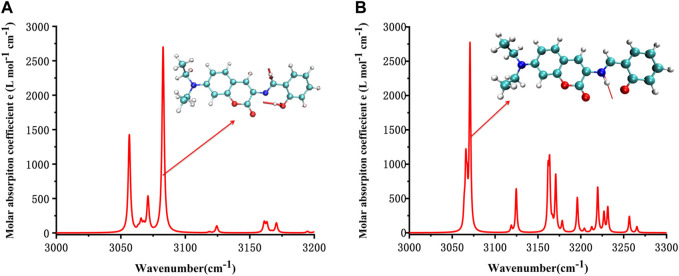
Simulated IR spectrum of **(A)** E1 and **(B)** K1.

Many kinds of interactions between adjacent atoms in the four probe molecules are depicted in Figure 3. It could be clearly inferred that the hydrogen bond interaction of N … O-H and N-H … O in E1 and K1 molecule respectively led to the much lower free energy and more stable structure compared with E2 and K2 molecules. E1 and K1 molecules were the most commonly found and used conformation for the biothiols probe according to reference 45. Therefore, only E1 and K1 probe molecules were considered for the electron excitation and emission process analysis. The clear difference between the structure of E1 and K1 molecules is intuitively shown in their simulated IR spectrum (Figure 4).

It can be seen from the simulated IR spectrum that O-H stretch vibration in E1 (3,081 cm^−1^, as shown by the red arrow both in Figure 4A and the inset E1 molecule) has a similar intensity to the N-H stretch vibration in K1 molecule (3,073 cm^−1^, as shown by the red arrow both in Figure 4B and the inset K1 molecule). But the different spectrum shape in this wavenumber range (3,000–3,300 cm^−1^) indicated the clear difference between the structure of E1 and K1 molecules.

**FIGURE 5:**
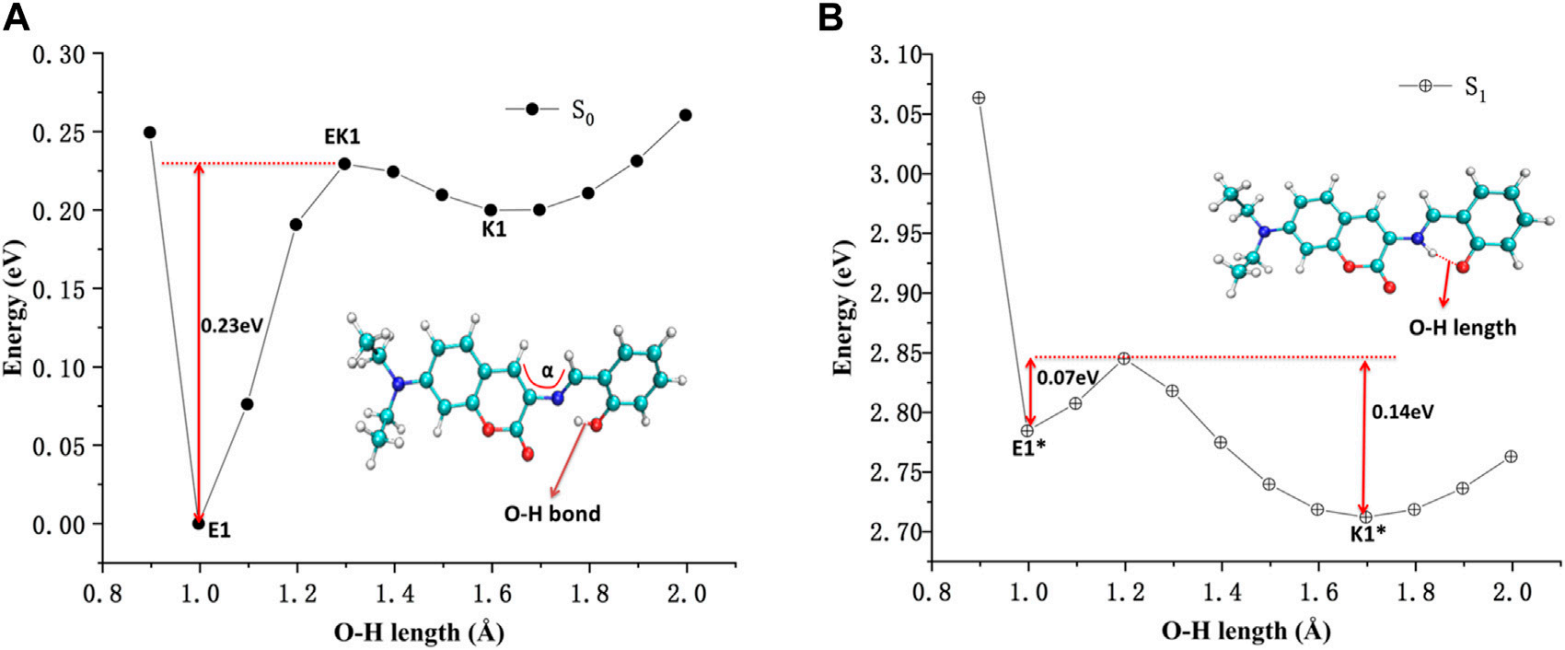
The potential energy curves of DEMCA-OH **(A)** ground state and **(B)** first excited state.

The potential energy curves of ground state and first excited state of DEMCA-OH are constructed in Figure 5. It can be clearly concluded that the Enol form (E1, O-H bond length with about 1.0 Å) was the most stable ground state (S_0_) for the DEMCA-OH, while the Keto* form (K1*, O-H bond length with about 1.7 Å and N-H bond length with about 1.0 Å) was the most stable first excited state (S_1_) for DEMCA-OH. Meanwhile, the energy barrier from E1* to K1* and E1 to K1 were 0.07 eV and 0.23eV respectively which led to the typical ESIPT process in the optical excitation of the DEMCA-OH. Dual emission band with different wavelengths corresponding to the E1* to E1 and K1* to K1 process could be obtained from this mechanism, which was consistent with the experimental results.

**FIGURE 6:**
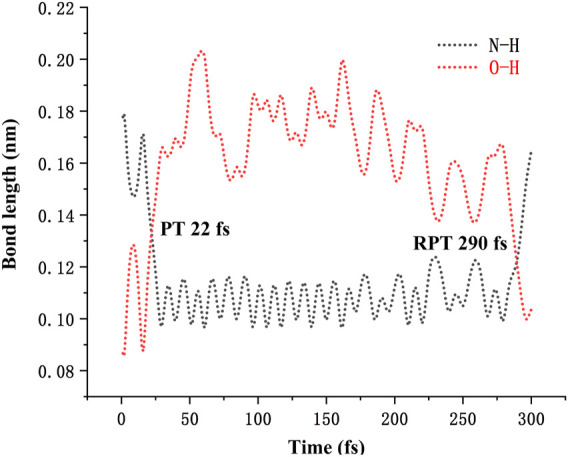
The variation of O-H and N-H bond length in S_1_ state of DEMCA-OH.

The energy surface hopping dynamics between the first excited state and ground state of DEMCA-OH was analyzed through the NX program. During the sufficient simulation time (300fs), the variation of O-H bond length was in the range of 0.8–2.1 Å. The proton transfer (PT, 22 fs) and reversed proton transfer (RPT, 290 fs) process shown in Figure 6 indicated the low energy barrier between the E1* and K1* state of probe DEMCA-OH. Meanwhile, the energy barrier from K1* to E1* (0.14 eV) was twice as much as the energy barrier from K1* to E1* (0.07 eV), which led to the longer duration in K1* over E1* as shown in the statistical results on the energy surface hopping dynamics of DEMCA-OH S_1_ state. The theoretical results were consistent with the larger measured fluorescent intensity with ESIPT emission (K1* to K1) over normal emission (E1* to E1), as shown in Figure 1 of reference [45].

**FIGURE 7:**
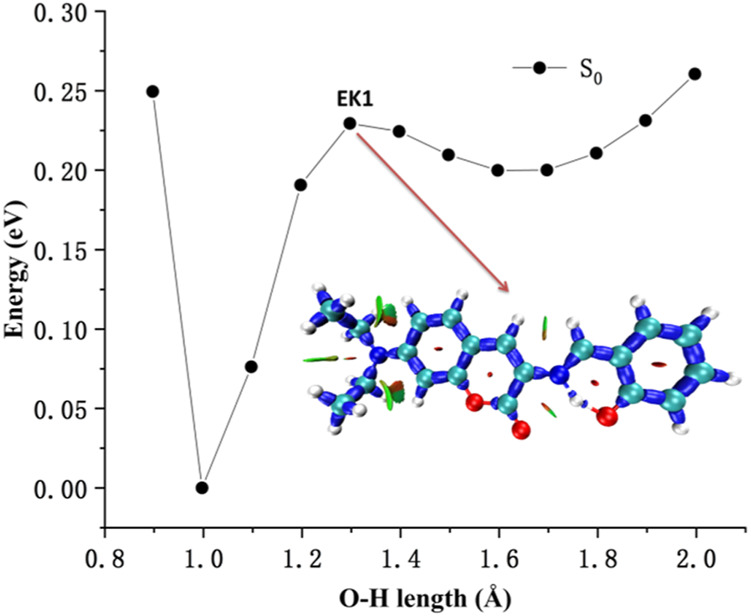
The interaction within transition state EK1.

**FIGURE 8:**
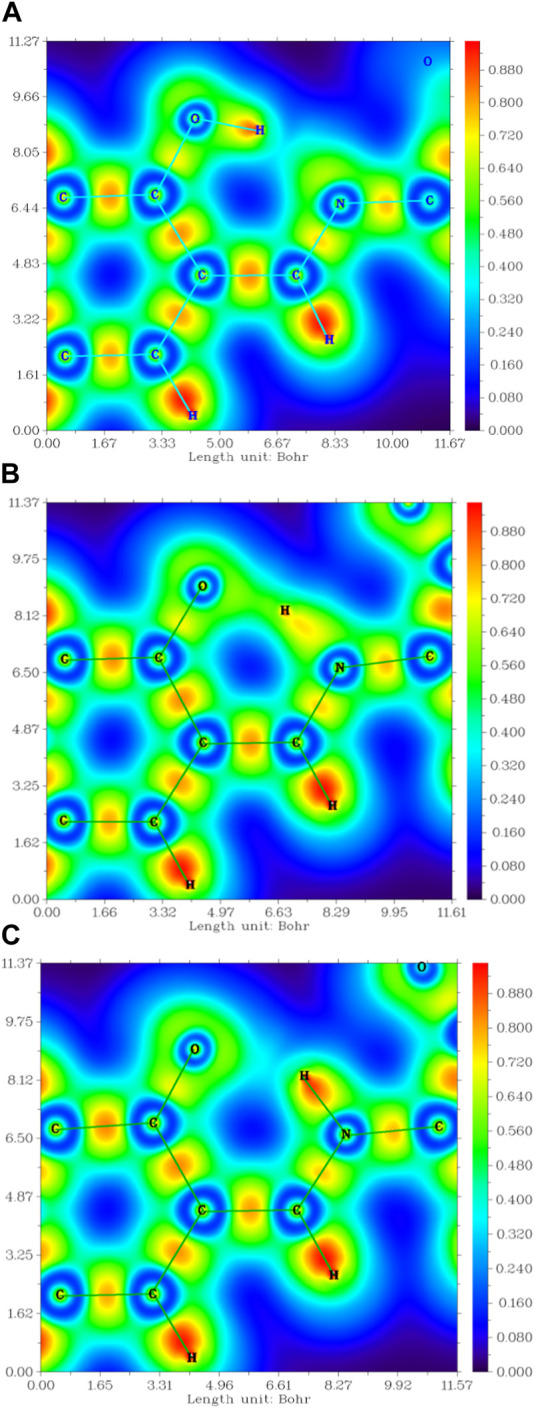
The 2D projection of the LOL in the N-H-O hydrogen bond area within **(A)** E1, **(B)** EK1, and **(C)** K1.

The interaction within transition state EK1 (O-H bond length 1.3 Å and N-H bond length 1.2 Å) from E1 to K1 was demonstrated in the potential energy curve of ground state S_0_ of DEMCA-OH, as shown in Figure 7. It was further confirmed that hydrogen bond interaction of N … O-H and N-H … O had a crucial role in the stability of the structure of DEMCA-OH. The 2D projection of the LOL in the N-H-O hydrogen bond area within E1, EK1, and K1 molecules is shown in Figure 8 and clearly indicates the electron distribution variation while the proton transfers between adjacent oxygen and nitrogen atoms. From Figures 7, 8, it can be concluded that competition between the strength of hydrogen bond interactions of N … O-H and N-H … O led to the stable molecular structure of DEMCA-OH. Due to the higher electric negativity of oxygen than nitrogen, the strength of O-H over N-H made E1 the preferable stable ground state structure of DEMCA-OH over K1.

**FIGURE 9:**
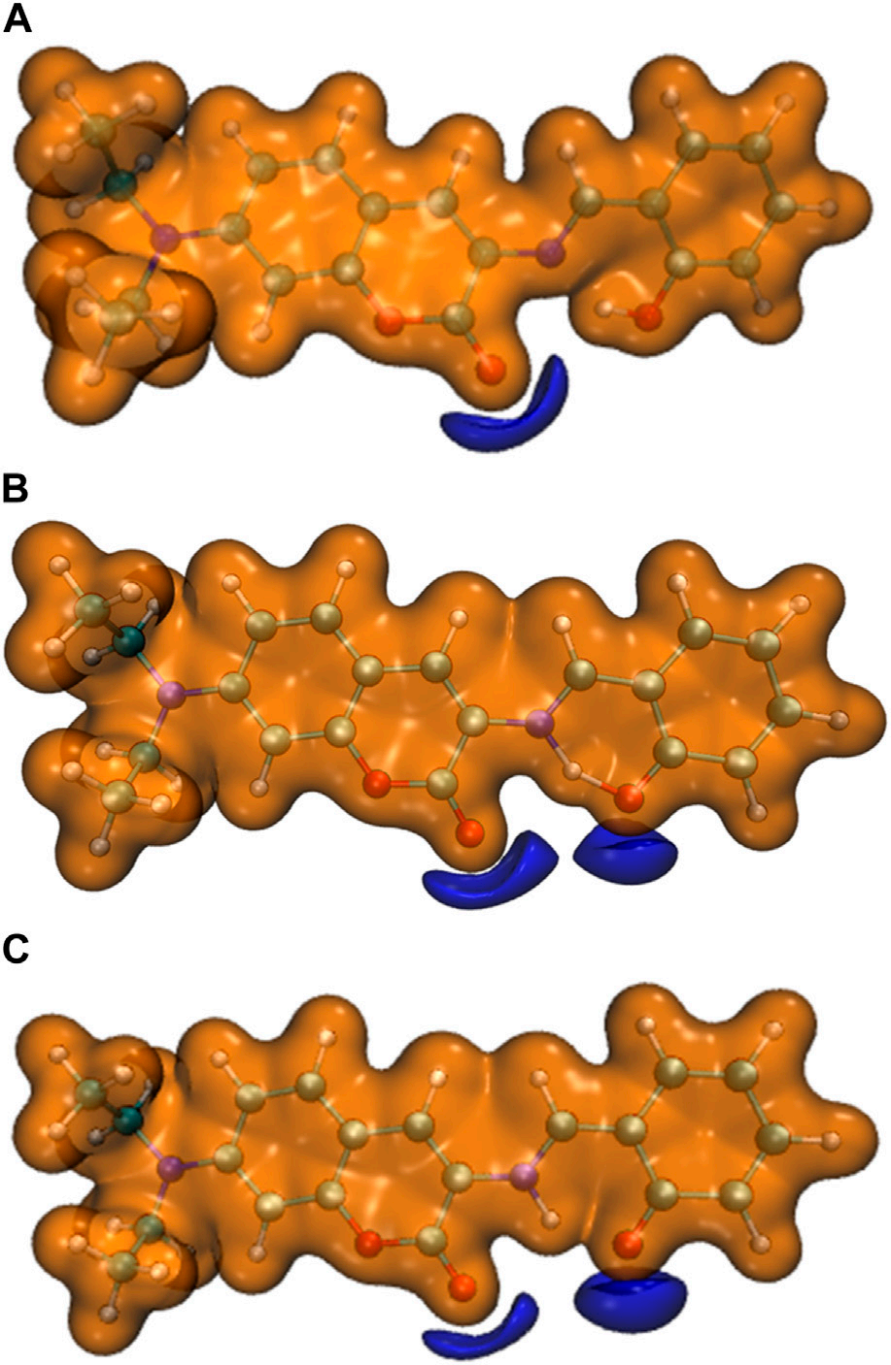
The static electrical potential of **(A)** E1, **(B)** EK1, and **(C)** K1 (orange: isovalue = 0.05 a.u. ,purple: isovalue = −0.05 a.u.).

Furthermore, the proton transfer also influenced the static electrical potential of the molecule, especially the neighbor area of the two adjacent oxygen atoms, as shown in Figure 9. It can be seen that when the O-H hydrogen bond was formed in E1, the shared electrons between oxygen and hydrogen reduced the absolute value of the static electrical potential near the oxygen atom. While the N-H hydrogen bond was formed in K1, the absolute value of the static electrical potential near the oxygen atom was clearly larger than that in the E1 molecule.

The main electron excitation and emission processes in the probe molecule DEMCA are summarized in Table 2 and Table 3 respectively. It can be seen that the main orbital composition correlated to the electron excitation and emission processes in the probes was between the highest occupied molecular orbital (homo) and lowest unoccupied molecular orbital (lumo). Compared with the E1 molecule, there were larger stoke shifts between the wavelengths of electron excitation and emissions within the K1 molecule, which was consistent with the experimental results (Ding et al., 2023b). Meanwhile, the oscillator strength of the electron transition from S_1_ to S_0_ in NBSC molecules was smaller than that within the E1 and K1 molecules, which was also found in the experimental results (Ding et al., 2023b).

**TABLE 2:** The main electron excitation processes in the probe molecule.

Probe	Electronic transition^a^	Excitation energy (nm)	Oscillator strength	Composition^b^	CI^c^
E1	S_0_ 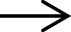 S_1_	438	0.9816	H 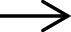 L	0.6815
K1	S_0_ 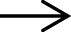 S_!_	443	1.0521	H 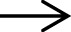 L	0.6908
NBSC	S_0_ 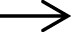 S_1_	421	0.0014	H 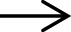 L	0.7103

^a^ Only the excited states with oscillator strengths larger than 0.1 were considered.

^b^ H stands for HOMO, and L stands for LUMO.

^c^ Coefficient of the wave function for each excitation was in absolute value.

**TABLE 3:** The main electron emission processes in the probe molecule.

Compound	Electronic transition^a^	Emission energy (nm)	Oscillator strength	Composition^b^	CI^c^
E1	S_1_ 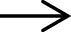 S_0_	485	0.9945	H 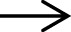 L	0.6726
K1	S_1_ 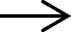 S_0_	567	1.1014	H 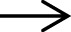 L	0.6017
NBSC	S_1_ 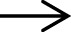 S_0_	493	0.0087	H 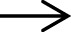 L	0.7015

a,b,c same indication as in Table 1.

**FIGURE 10:**
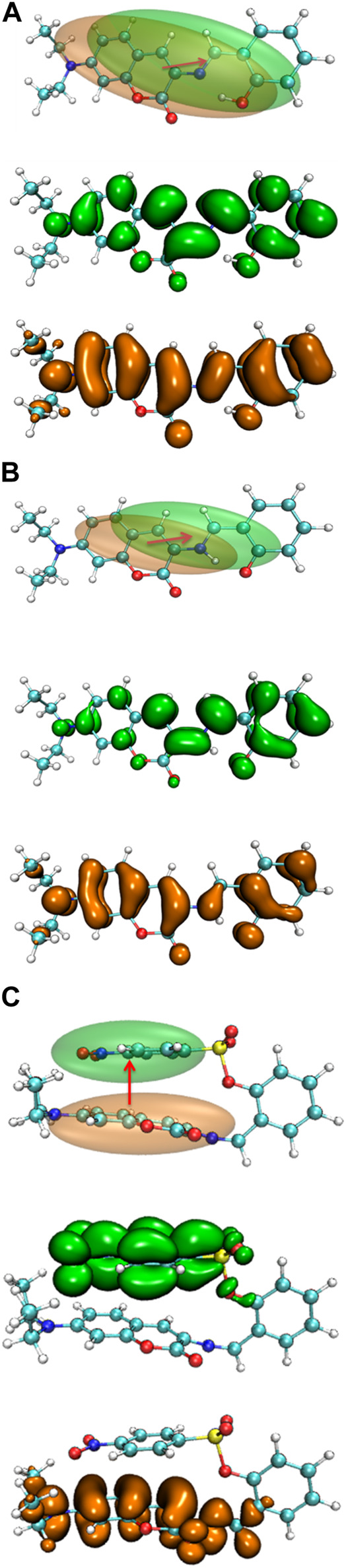
The electron density difference between the S_0_ and S_1_ of **(A)** E1, **(B)** K1, and **(C)** DEMCA-NBSC.

The electron density difference between the S_0_ and S_1_ of E1 and K1 are shown in Figure 10. The electrons were excited from the hole area (orange part) to the electron area (green part). The absorption and emission bands in E1 and K1 could be due to the (π,π) transition with intramolecular local charge transfer character. While the corresponding absorption and emission bands in NBSC should be produced by a non-local charge transfer process, as shown in Figure 10C.

**FIGURE 11:**
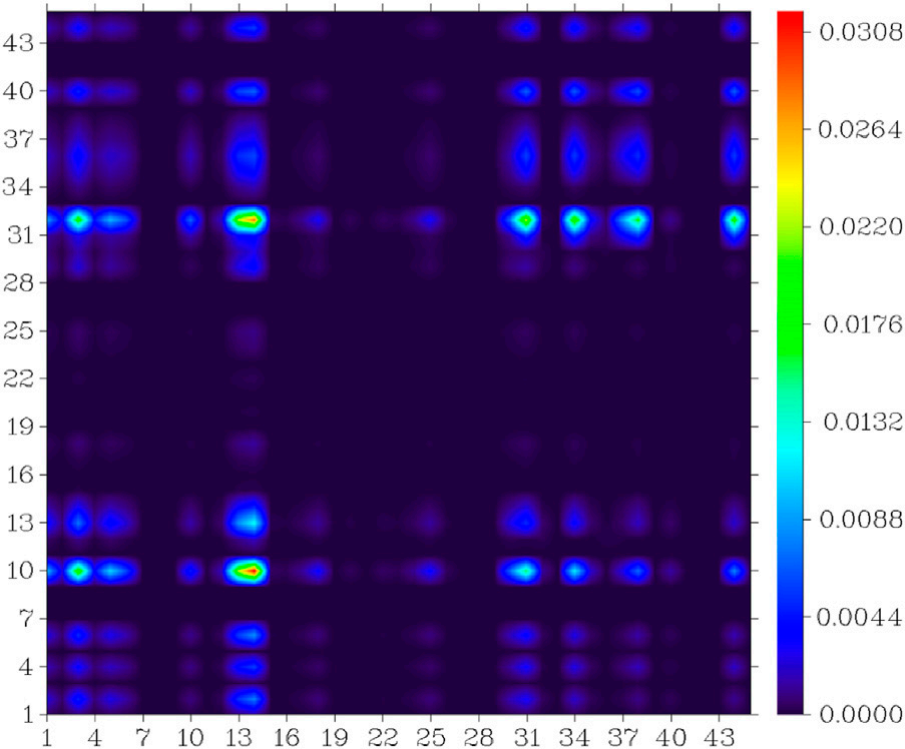
Atom-atom electron transfer heat map of the E1 molecule from S_0_ to S_1._

**FIGURE 12:**
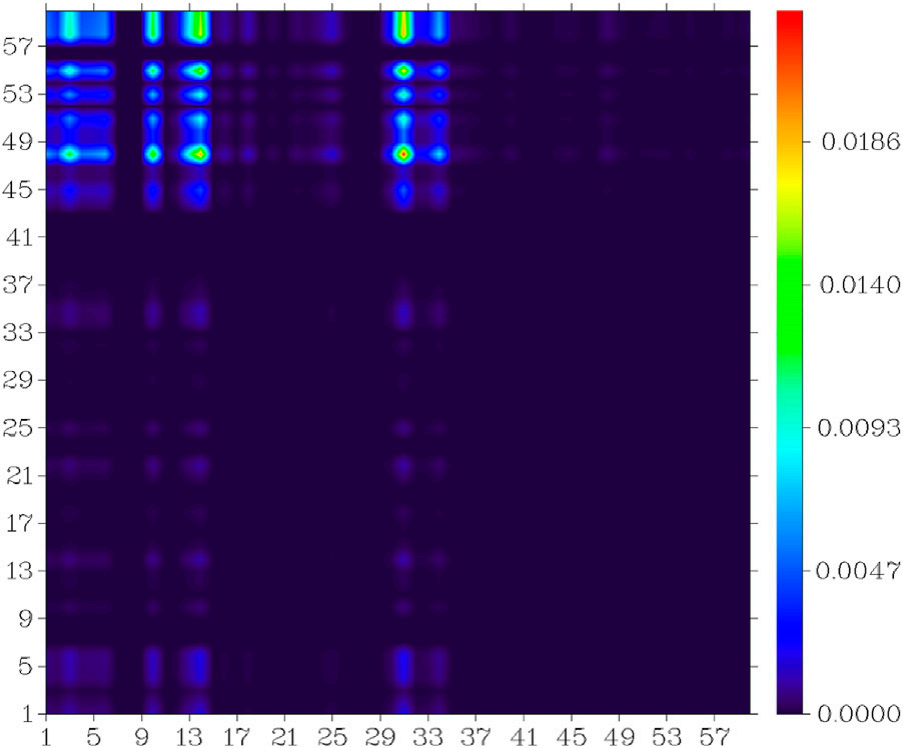
Atom-atom electron transfer heat map of the DEMCA-NBSC molecule from S_0_ to S_1._

In the S_0_ to S_1_ electron excitation process, the biggest electron donor and acceptor atoms were N (No.14) and C (No.10 and No.32) respectively (atom numbers were referred to Supplementary Figure S2), which was indicated in the atom-atom electron transfer heat map of the E1 molecule (Figure 11). Two end ethylene groups (atom No.15-No.28) were hardly involved in this process, which is indicated in Figure 11 as well. For clarity, the similar atom-atom electron transfer heat map of the K1 molecule with the E1 molecule was not depicted here. The atom-atom electron transfer heat map of DEMCA-NBSC molecule (Figure 12, atom numbers are referred to in Supplementary Figure S3) clearly indicated the non-local intra charge transfer character from S_0_ to S_1_ electron excitation process just as the results mentioned before.

The theoretical and experimental center wavelengths of the absorption and emission bands of the probes are summarized in Table 4. Although there was a clear deviation from the experimental wavelength, a similar trend of wavelength varying with the experimental value was found in the calculation. It can be seen that there are larger stoke shifts between absorption and emission wavelengths in the K1 molecule than the E1 molecule from both the theoretical and experimental results.

**TABLE 4:** The calculated [experimental (Ding et al., 2023b)] excitation and emitting fluorescence wavelength of the probes.

Probe	λ_ex_ (wB2GP-PLYP in DMF, nm)	λ_ex_ (experimental in DMF, nm)	λ_em_ (wB2GP-PLYP in DMF, nm)	λ_em_ (experimental in DMF, nm)
E1	438	450	485	500
K1	443	450	567	575
NBSC	421	430	493	500

## 4 Conclusion

The different stable conformations of probe DEMCA-OH were found through a quantum mechanical method. The E1 and K1 form received lower energy due to the hydrogen bond interaction which stabilized the structure of the probe. The low energy barrier between their first excited state E1* and K1* indicated the typical ESIPT process within the optical excitation on the probe DEMCA-OH. The energy surface hopping dynamics analysis on the first excited state further confirmed that the ESIPT process happened in the electronic excitation of the probe DEMCA-OH. Meanwhile, the electron density difference analysis between the ground state and first excited state indicated a non-local intramolecular charge transfer process in DEMCA-NBSC rather than a local intramolecular charge transfer process (π,π transition), which occurred in DEMCA-OH when the molecules were under optical excitation. All these results provide insights into preparing a wide range of fluorescent probes and expanding the potential for widespread use in bio-medical applications.

## Data Availability

The original contributions presented in the study are included in the article/Supplementary Material, further inquiries can be directed to the corresponding author.
