# Supplementary material for: Theoretical investigation on a simple turn on fluorescent probe for detection of biothiols based on coumarin unit
Source: Front Chem. 2023 Nov 8;11:1290745. doi: 10.3389/fchem.2023.1290745 (PMC10663294; doi:10.3389/fchem.2023.1290745)
Supplement: Supplementary file 1 [file DataSheet1.docx]

**Theoretical investigation on a simple turn on fluorescent probe for biothiols detection based on coumarin unit**

Tianhao Ma^1^, He Huang^2^, Yuling Liu^2^, Yongjin Peng^2*^

1 Affiliated 3rd hospital, Jinzhou Medical University, Jinzhou 121001, P. R. China

2 College of Bio-informational Engineering, Jinzhou Medical University, Jinzhou 121001, P. R. China


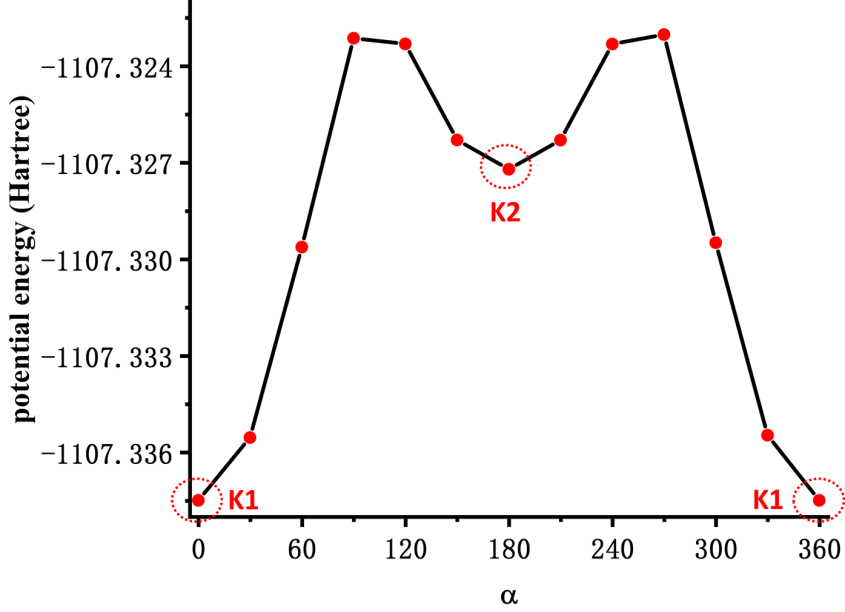


Figure S1 The potential energy scan curve of DEMCA-OH on α


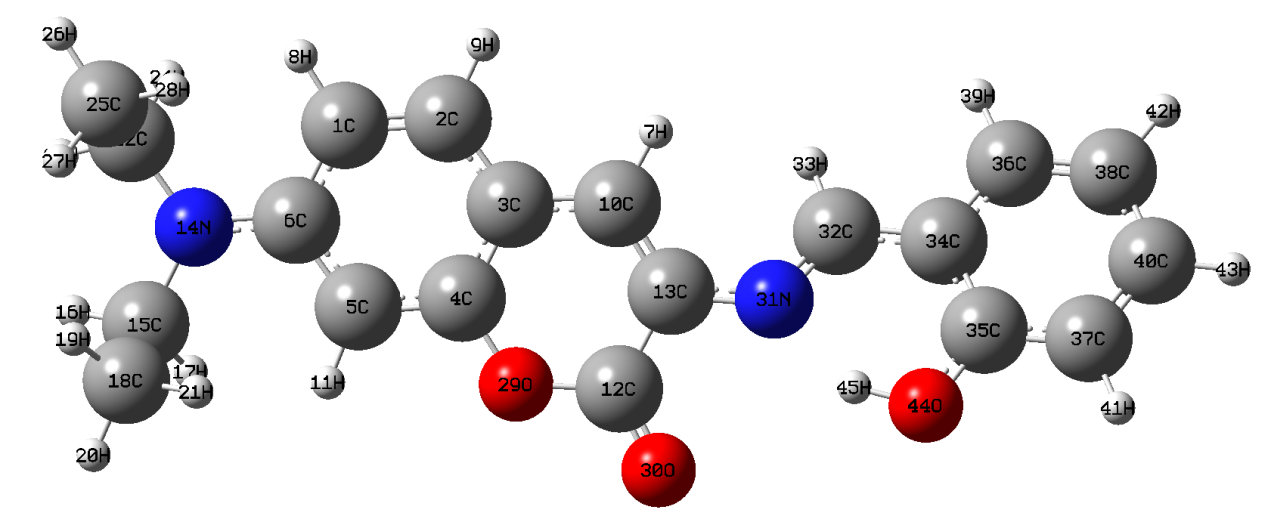


Figure S2 the stable structure of E1


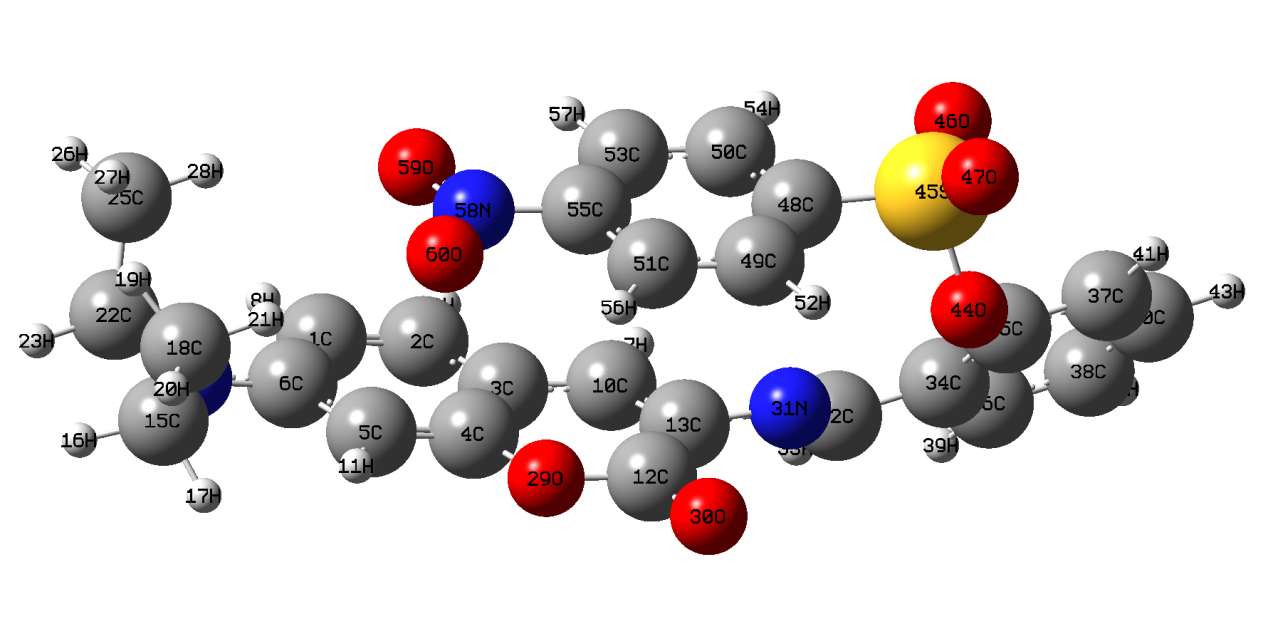


Figure S3 the stable structure of DEMCA-NBSC

(Dr Min Feng from Nankai University was appreciated for using Gaussview to make Figure S2 and S3)
